# Supplementary material for: Treadmill belt accelerations may not accurately replicate kinematic responses to tripping on an obstacle in older people
Source: PLoS One. 2025 Jan 9;20(1):e0312261. doi: 10.1371/journal.pone.0312261 (PMC11717211; doi:10.1371/journal.pone.0312261)
Supplement: S1 File — (DOCX) [file pone.0312261.s001.docx]

**Appendix A. Detailed methods for biomechanical parameters**

Marker trajectories were filtered with a 10 Hz low-pass fourth order (zero lag) Butterworth filter. Custom MATLAB scripts (R2019b, The MathWorks, Inc., MA, USA) were used to calculate kinematic variables for one previous (Pre) and five recovery steps (Post1, Post2, Post3, Post4, Post5) after trip-onset. On the walkway, regardless of the recovery strategy, step Post1 initiated with the first foot-strike of the obstructed non-dominant left foot and ended with the following foot-strike by the contralateral dominant right foot. On the treadmill, step Post1 initiated with the foot-strike of the non-dominant left foot on the accelerated belt and ended with the following foot-strike of the unperturbed dominant right leg. Dynamic stability was quantified by the anteroposterior (AP) margin of stability (MoS) at the foot-strike of each step. MoS was defined as the distance (cm) between the closest AP border of the base of support (BoS) (often the leading foot toe) and the extrapolated centre of mass position (${xCoM}_{P}$) ^1^; ^2^, calculated as follows:

$$x{CoM}_{P}={CoM}_{P} +\frac{{CoM}_{v}+{BoS}_{\bar{v}}}{\sqrt{\frac{g}{L}}}$$

where ${CoM}_{P}$ refers to the $CoM$ position, ${CoM}_{v}$ is the $CoM$ velocity, ${BoS}_{\bar{v}}$ is the $BoS$ velocity (i.e. averaged heel marker velocity during stance on the treadmill and zero on the walkway), $g$ the gravitational acceleration (9.81 m s^-2^) and $L$, the distance between the $CoM$ and the leading foot ankle marker in the sagittal plane. Positive MoS signifies that an individual’s body is in a state of stable balance ($xCoM$ is within the BoS), while negative MoS indicates body instability ($xCoM$ is outside the BoS).

The magnitude of a perturbation was quantified as the $xCoM$ position at each foot-strike relative to the position of the ankle marker at the previous foot-strike (cm). A larger $xCoM$ value signifies a greater anterior progression of $xCoM$ during the step, thus indicating a greater magnitude of perturbation. The maximum AP velocity of $CoM$ (m s^-1^), step length (cm), maximum toe clearance (cm), and maximum trunk, hip and knee angles (°) during each step were also derived. In addition, a hop which required rapid force production was defined when both feet were off the floor during the step. See Supplementary Table 1 for more details.

For the fifth and sixth unperturbed walking trials, average MoS (cm), gait speed (m s^-1^), cadence (steps min^-1^) and step length (cm) were calculated, and the results pooled for analysis of unperturbed gait characteristics.

Missing foot marker and CoM data that could not be computed by the standard gap filling procedure in Vicon Nexus were estimated using a custom MATLAB code. Following MATLAB processing and parameterisation, all data were exported into Excel (Microsoft, Redmond, WA, USA) for visual identification and verification of outliers. Outliers reflecting genuine behaviours were retained, but eliminated if gap filling or estimation were unsuccessful. See Appendix B for the employed data handling procedure.

**Supplementary Table 1** | Definitions of biomechanical parameters related to the treadmill and walkway.

| Parameter | Condition | Definition |
| --- | --- | --- |
| ***Unperturbed walks*** | | |
| Step length (cm) | Walkway | Average of anteroposterior travel distance from a lateral malleoli ankle marker at a foot-strike to the lateral malleoli ankle marker of the next foot-strike. When the ankle marker of the next foot-strike (trailing foot) does not surpass the position of the ankle marker of the previous foot-strike (leading foot), the step length would be negative. |
|  | Treadmill | A step length on the treadmill was estimated as the average step time (s) times average belt speed (cm/s) while adjusting for the difference in foot positions (ankle markers) (cm). When the ankle marker of the next foot-strike (trailing foot) does not surpass the position of the ankle marker of the previous foot-strike (leading foot), the step length would be negative. |
| Cadence (steps/min) | Walkway and Treadmill | Derived from the number of steps taken during a trial divided by time, transformed to steps per minute. |
| Gait velocity (m/s) | Walkway | Calculated from the difference in anteroposterior position of the centre of mass between the first and last foot-strike over time, transformed to m/s. |
|  | Treadmill | Calculated using anteroposterior velocity of left and right heel markers during stance according to Souza, et al. ^3^. |
| ***Events in trip trials*** |  |  |
| Previous step | Walkway | The last unperturbed step which ends with the last foot-strike before trip onset. |
|  | Treadmill | The last unperturbed step which ends with the foot-strike at perturbation onset. |
| Trip onset | Walkway | The moment in time when the foot first contacts the tripping obstacle during swing phase. |
|  | Treadmill | The moment in time when the foot strikes the treadmill belt that has accelerated. |
| Recovery steps | Walkway | The first recovery step (Post1) begins with the last foot-strike before trip onset and ends with the 1^st^ foot-strike after trip onset. The second recovery step (Post2) begins with the 1^st^ foot-strike after trip onset and ends with the 2^nd^ foot-strike after trip onset. The subsequent steps were defined as third (Post3), fourth (Post4) and fifth (Post5) recovery steps in the same manner. |
| Recovery steps | Treadmill | Post1 begins with the perturbation onset (1^st^ foot-strike) and ends with the 2^nd^ foot-strike after perturbation onset. Post2 begins with the 2^nd^ foot-strike and ends with the 3^rd^ foot-strike after the perturbation onset. The subsequent steps were defined as Post3, Post4 and Post5 in the same manner. |
| ***Kinematics in trip trials*** | | |
| Stepping strategy | Walkway | Determined by the anteroposterior position of the ankle marker of the perturbed leg relative to the obstacle at time of first foot-strike after trip onset. If the ankle marker remains posterior to the obstacle, then the trial is classified as lowering. If the ankle marker is anterior to the obstacle, then the trial is classified as elevating. |
| Hop | Walkway and Treadmill | A hop during each step was defined when both feet were off the floor during the step. |
| Fall | Walkway and Treadmill | A fall during the trial was defined when a load cell, situated in series with the body harness and its anchor point on the ceiling rail, recorded >30% of the participant’s body weight following a perturbation ^4^. |
| Extrapolated CoM (xCoM) | Walkway and Treadmill | The extrapolated centre of mass position (${xCoM}_{P}$) ^1^; ^2^ was calculated as follows:  $x{CoM}_{P}={CoM}_{P} +\frac{{CoM}_{v}+{BoS}_{\bar{v}}}{\sqrt{\frac{g}{L}}}$  where ${CoM}_{P}$ refers to the $CoM$ position at a foot-strike, ${CoM}_{v}$ is the average $CoM$ velocity, ${BoS}_{\bar{v}}$ is the average $BoS$ velocity (i.e. averaged heel marker velocity during stance on the treadmill and zero on the walkway), $g$ the gravitational acceleration (9.81 m s^-2^) and $L$, the distance between $CoM$ and the leading foot ankle marker in the sagittal plane. So far ${xCoM}_{P}$ has been calculated as the global position in the lab space. To quantify the magnitude of perturbation caused by the trip, we calculated the $xCoM$ during each step as the distance between ${xCoM}_{P}$ at each foot-strike relative to its previous foot-strike ankle position (cm). A larger $xCoM$ value signifies a greater anterior progression of $xCoM$ during the step. |
| Margin of stability (MoS) (cm) | Walkway and Treadmill | Calculated as difference in anteroposterior position of xCoM (${xCoM}_{P}$) and anteroposterior border of base of support (BoS). The anterior border of BoS was defined as the front edge of the leading foot in the sagittal plane, taken as the toe marker (situated on the second metatarsal head) plus 30% of the anteroposterior distance between the toe and heel markers (to account for the anterior boundary of the shoe). The posterior border of BoS was defined as the heel marker position of the trailing foot. The anterior or posterior border of BoS, whichever is closest to the ${xCoM}_{P},$ was used to calculate the MoS. If the ${xCoM}_{P}$ is outside of the BoS, then the MoS will have a negative value to denote an unstable state. If the ${xCoM}_{P}$is inside the BoS, then the MoS will have a positive value to denote a stable state.  The MoS was calculated at the foot-strike of each step (Pre, Post1-5). As each step starts with a foot-strike and concludes with another foot-strike, the concluding foot-strike was used to label the MoS. |
| Maximum CoM anteroposterior velocity (m/s) | Walkway | Maximum velocity of the CoM in the anteroposterior direction within each step (m/s). |
|  | Treadmill | Maximum velocity of the CoM (CoM velocity + belt velocity) in the anteroposterior direction within each step (m/s). |
| Maximum toe clearance | Walkway and Treadmill | Maximum vertical difference in height of the swing foot toe marker and the stance foot toe marker within each step. |
| Maximum trunk angle (deg) | Walkway and Treadmill | Peak flexion angle of the sagittal plane trunk angle within each step. |
| Maximum hip angle (deg) | Walkway and Treadmill | Peak flexion angle of the sagittal plane hip angle within each step (swing leg). |
| Maximum knee angle (deg) | Walkway and Treadmill | Peak flexion angle of the sagittal plane knee angle within each step (swing leg). |

**References**

1. Hof A, Gazendam M, Sinke W. The condition for dynamic stability. *Journal of Biomechanics* 2005;38(1):1-8.

2. Süptitz F, Catalá MM, Brüggemann G-P, et al. Dynamic stability control during perturbed walking can be assessed by a reduced kinematic model across the adult female lifespan. *Human Movement Science* 2013;32(6):1404-14.

3. Souza GSdSe, Rodrigues FB, Andrade AO, et al. A simple, reliable method to determine the mean gait speed using heel markers on a treadmill. *Computer Methods in Biomechanics and Biomedical Engineering* 2017;20(8):901-04. doi: 10.1080/10255842.2017.1309395

4. Yang F, Pai Y-C. Automatic recognition of falls in gait-slip training: harness load cell based criteria. *Journal of Biomechanics* 2011;44(12):2243-49.

5. Abdi H. Holm’s Sequential Bonferroni Procedure. In: Salkind N, ed. Encyclopedia of Research Design. Thousand Oaks, CA: Sage 2010.

**Appendix B. Data Handling Procedure**

**1. Vicon Nexus 2**

1. Trial data collected in Nexus. Reconstruct 3D marker trajectories.
2. Gap filling procedure (starting from a pipeline then manually fixing remaining gaps and other issues)
3. Woltring (max gap length=5 frames) *1 frame = 10 ms
4. Cyclic (max gap length=25 frames)
5. Rigid body (max gap length=1000 frames) requires three reference markers/trajectories on the same segment
6. Pattern fill (max gap length=100 frames) requires one reference marker/trajectory
7. Filter trajectories using a Butterworth low-pass (10Hz) zero lag fourth order filter.
8. Process Dynamic Plug-in Gait Model to estimate CoM and trunk (thorax), hip and knee angles.
9. Export the trial data as c3d files.

**2. MATLAB**

1. Import the c3d files into MATLAB.
2. Estimate
   1. The missing centre of mass (CoM) trajectory based on its relative distance from the available pelvis markers (left/right ASIS and PSIS). The CoM trajectory is usually present before a perturbation onset and absent after the perturbation and towards the end of the trial. During the frames when CoM was present, its distance between the four pelvis markers were calculated. When the CoM was absent, its trajectory was estimated based on the available markers.
   2. The missing foot marker trajectory based on its relative distance from the available foot markers on the same segment (LTOE/LANK/LHEE and RTOE/RANK/RHEE).
3. Calculate and export the parameters of interest into CSV files.

**3. Data Verification / Excel spreadsheet**

1. Examination of outliers using a scatter plot for each variable.
2. The following data was eliminated; Missing data that could not be properly estimated e.g. the clavicle marker was obscured by the harness or shirt for the whole duration of the trial. Data elimination was conducted by segment. Outliers due to unusual but genuine behaviors were retained in the dataset e.g. the participant loosing balance following a trip.
3. Outliers due to mistakes were corrected (e.g. incorrectly labelled markers).

**4. Statistical analyses**

1. Import the CSV file into SPSS.
2. Restructure the data into long-format for the generalised linear mixed-effects model (GLMM) analysis.
3. Run the GLMM.
4. Examine the QQ plot to examine the normality and model fit.
5. Transform the data with Log 10 if needed.
6. Run the GLMM.
7. Compare the QQ plots for the crude and log-transformed data and select the model with better model fit.
8. To control for the family-wise error rate, unadjusted P values obtained from the GLMM were corrected with the Holm-Bonferroni method using the following formula

$$P\left( adjusted \right)=P\left( C-i+1 \right),$$

where *C* is the number of tests (6 steps [tests] x 2 models = 12) and *i* is the rank of *P* values among the tests (e.g. 1 for the smallest *P* value) ^5^

**Number of trials that required missing data estimation**

|  | Treadmill | Walkway-Elevating | Walkway-Lowering |
| --- | --- | --- | --- |
| The missing CoM trajectory was estimated for at least one frame (10 ms) | 21.2%, 14/66 trials | 79.1%, 19/24 trials | 78.4%, 29/37 trials |
| The missing foot trajectory was estimated for at least one frame (10 ms) | 0%, 0/0 trials | 37.5%, 9/24 trials | 56.8%, 21/37 trials |

The CoM trajectory created by the Vicon Nexus Plug-in-Gait model requires all markers to be present, thus absence of at least one maker resulted in a missing CoM trajectory during the affected frames. Missing marker trajectories occurred due to (1) the participant moving into the suboptimal capture space on the 10 m walkway (only the middle 4 m is captured by all eight cameras), (2) occlusion of marker(s) from the camera sight due to large trunk flexion, (3) loss of the physical marker due to impact of collision to the trip-board, rapid arm and leg movements (that sometimes physically knocked a marker off e.g. the right swinging arm knocking the right thigh marker) or sweat. The 0% for missing foot trajectories in the treadmill trials means that all gaps in the foot marker trajectories were able to be filled with the gap filling procedure within Vicon Nexus. The missing CoM trajectory was estimated based on its relative distance from the available pelvis markers (left/right ASIS and PSIS). The CoM trajectory is usually present before a perturbation onset and disappear after the perturbation or towards the end of the trial. During the frames when CoM was present, its distance between CoM and the four pelvis markers were calculated. When the CoM disappeared, its trajectory was estimated based on the available pelvis markers.

**Number of datapoints eliminated during data verification**

|  | Treadmill | Walkway-Elevating | Walkway-Lowering |
| --- | --- | --- | --- |
| Step Length | 0%, 0/396 datapoints | 0.69%, 1/144 datapoints | 0%, 0/259 datapoints |
| Max. Toe Clearance | 0%, 0/396 datapoints | 0%, 0/144 datapoints | 0%, 0/259 datapoints |
| Max. Knee Angle | 0%, 0/396 datapoints | 0.69%, 1/144 datapoints | 1.16%, 3/259 datapoints |
| Max. Hip Angle | 0%, 0/396 datapoints | 0.69%, 1/144 datapoints | 1.16%, 3/259 datapoints |
| Max. Trunk Angle | 0%, 0/396 datapoints | 1.39%, 2/144 datapoints | 1.54%, 4/259 datapoints |
| Max. CoM AP Vel | 0%, 0/396 datapoints | 1.39%, 2/144 datapoints | 4.63%, 12/259 datapoints |
| xCoM | 0%, 0/396 datapoints | 2.08%, 3/144 datapoints | 0%, 0/259 datapoints |
| MoS | 0%, 0/396 datapoints | 2.08%, 3/144 datapoints | 0.39%, 1/259 datapoints |
| Total eliminated: | 0%, 0/396 datapoints | 1.02%, 13/1272 datapoints | 1.00%, 23/2294 datapoints |

|  | Treadmill Normal | Walkway Normal |
| --- | --- | --- |
| MoS | 0%, 0/0 datapoints | 1.37%, 1/73 datapoints |
| Cadence | 0%, 0/0 datapoints | 0%, 0/0 datapoints |
| Gait Speed | 0%, 0/0 datapoints | 0%, 0/0 datapoints |
| Step Length | 0%, 0/0 datapoints | 0%, 0/0 datapoints |
| Total eliminated: | 0%, 0/0 datapoints | 0.34%, 1/292 datapoints |


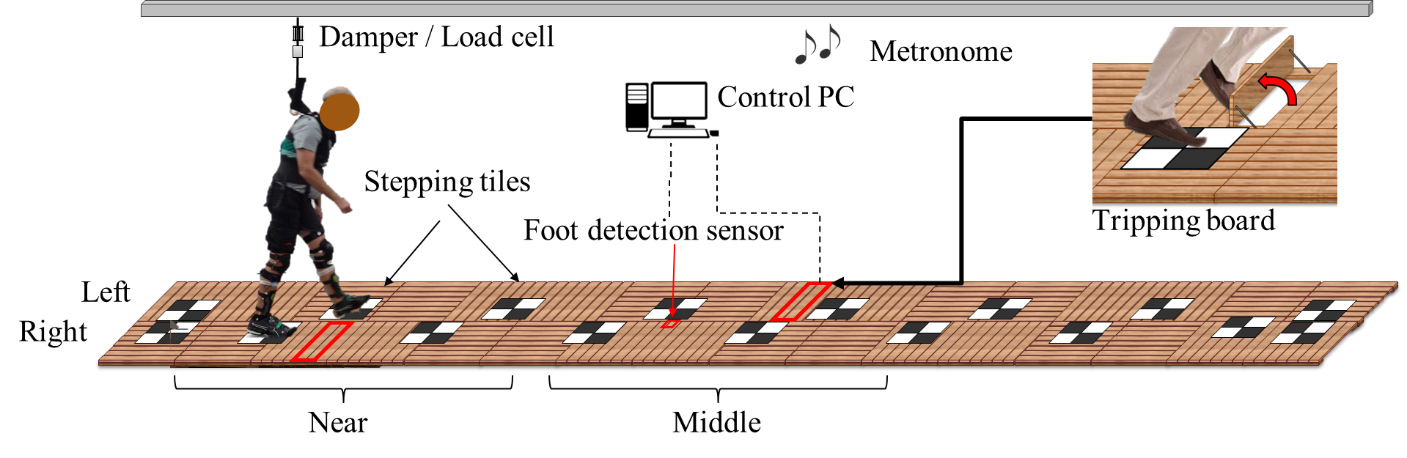


**Supplementary Figure 1** | The walkway used for inducing trips.

The first trip was induced at the middle of the walkway to the left foot during the mid- to late- (≥50%) swing phase. The second trip was induced at the near part of the walkway to the right foot during the early- to mid- (<50%) swing phase. Participants were instructed to step on the black/white stepping tiles in time with the beat of the metronome (individually set to 90% of their usual gait speed) to ensure consistent walking speed.


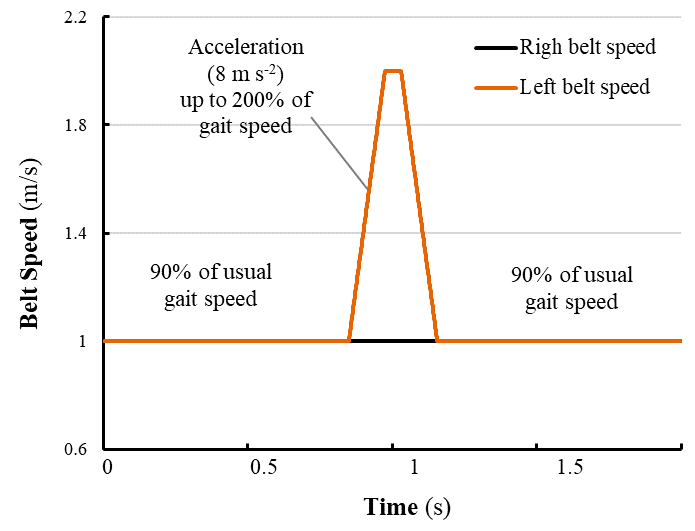


**Supplementary Figure 2** | The property of the treadmill belt acceleration.

The perturbation was applied to the non-dominant left limb via treadmill belt acceleration at 8 m s^-2^ to a maximum of 200% of usual walking speed for 30% of stride time. Perturbations were triggered when the hallux motion capture marker of the ipsilateral swing foot as it passed the hallux marker of the contralateral limb in the sagittal plane.
