# Supplementary material for: Treadmill belt accelerations may not accurately replicate kinematic responses to tripping on an obstacle in older people
Source: PLoS One. 2025 Jan 9;20(1):e0312261. doi: 10.1371/journal.pone.0312261 (PMC11717211; doi:10.1371/journal.pone.0312261)
Supplement: Attachment — Submitted filename: Cathy_Response letter V3 30May2024.docx [file pone.0312261.s002.docx]

Dear Editor and Reviewers,

We greatly appreciate the time and effort you have dedicated to providing constructive feedback and valuable suggestions. Each of your comments has been carefully considered, and we have implemented revisions to address the concerns raised. Below, we present a point-by-point response to each comment. We feel that the clarity of the manuscript has improved significantly following the revision and look forward to receiving further comments.

Sincerely,

Dr Yoshiro Okubo

On behalf of the authors

**Reviewer: 1**

Comments to the Author

1. Thank you for the opportunity to peer review. One primary finding is that perturbations from treadmills dissipate rapidly after the second recovery step, whereas those from overground walking persist across multiple recovery steps. In addition, they found treadmill accelerations induced smaller trunk displacement, step lengths, toe clearance and no falls than overground walking. This research is invaluable in terms of addressing the validation of treadmill accelerations for training of trip. This research is valuable for physical therapists. However, please respond to some revisions to improve the clarity of the article.

[Author response] Thank you for the positive and constructive feedback, which has greatly helped us improve the clarity of this manuscript.

2. p.5 Line 5: Please clarify which tasks are being compared in this study. In the introduction, the authors mentioned: "Trips during gait have been simulated on a treadmill by applying blocks to the treadmill surface [24]; [25], backward ankle-cable pulls [26]; [27], rapid belt accelerations on foot strike [28]; [14] or a combination of rapid belt accelerations and decelerations [29] ". However, it remains unclear which specific task was selected for this study, thereby the basis of the hypothesis questionable.

[Author response] We have edited the introduction to clarify that we intended to compare treadmill belt accelerations to obstacle-induced trips on a walkway as follows:

*“Compared to other methods involving block obstacles on the treadmill surface [26]; [27], or backward ankle-cable pulls [28]; [29] treadmills that can induce rapid belt accelerations during gait [30]; [14] and are easier to administer. Treadmill belt accelerations have been associated with reduced dynamic stability during the first recovery step [14] and sudden forward trunk flexion [24], which are also seen following obstacle-induced trip responses [26]; [29].” (page 5, lines 3-8)*

3. p.5 Line 12: Please specify standard of "validity". I could not understand what would be considered a valid proof in this study.

[Author response] Kinematic validity referred to the degree of similarity between kinematic responses induced by treadmill belt accelerations and obstacle-induced trips on the walkway. This has now been clarified as follows:

*“No previous studies have directly contrasted the kinematic responses induced by treadmill belt accelerations to those induced by obstacle-induced trips while walking on a walkway [15].” (page 5, lines 10-12)*

4. p.5 Line 15: Similar to the above, the "similar" is ambiguous; please specify further. Focusing on the adequacy of the perturbation magnitude within the surrogate task, as a training stimulus, would enhance clarity.

[Author response] We have now replaced the term “similar” with “the same degree” and the adequacy of the acceleration properties has been addressed as follows:

*“Using maximum acceleration properties we considered to be safe for older adults, we tested the following hypotheses: (1) While treadmill belt accelerations may initially destabilise balance (i.e. a negative margin of stability [MoS]) to the same magnitude as walkway trips (no statistically significant difference), such destabilisation would quickly dissipate following the first recovery step only on the treadmill [14].” (page 5, lines 16-20)*

5. p.5: Please include a detailed description of the specific kinematic characteristics of the selected task. I could not understand the foundational knowledge that would validate the hypothesis is not well understood.

[Author response] We have now included kinematic parameters we have investigated in our hypotheses. For more detailed descriptions of the specific kinematic variables, please refer to the methods section.

*(1) While treadmill belt accelerations may initially destabilise balance (i.e. a negative margin of stability [MoS]) to the same magnitude as walkway trips (no statistically significant difference), such destabilisation would quickly dissipate following the first recovery step only on the treadmill [14]. (2) Maximum trunk flexion following treadmill and walkway perturbations would be similar, but (3) step kinematics (toe clearance and step length) and lower-limb joint angles (maximum hip and knee flexion angles) following treadmill belt accelerations would be smaller compared to walkway trips [15].” (page 5, lines 17-23)*

6. p.7 Line 8: Please specify the number of perturbation trial in both overground and treadmill walking. Readers cannot understand the number of perturbation trial until they read the analysis.

[Author response] For both walkway and treadmill conditions, we administered five unperturbed walking trials, then two perturbation trials with another unperturbed walking trial in between. This has now been clarified as follows:

*“We administered five unperturbed walking trials (each for 10 m) followed by two semi-random trip trials separated by at least one unperturbed walking trial to ensure no predictive behaviours.” (page 7, lines 9-11)*

*“We administered five unperturbed walking trials (each lasting 30 s) followed by two semi-randomly presented perturbation trials separated by at least one unperturbed walking trial.” (page 7, lines 23-25)*

7. p.9 Line 20: Please describe this information by gender.

[Author response] We have now stratified the participant demographic data by gender in Table 1 as follows:

***Table. 1****. Key participant demographics and baseline spatiotemporal parameters of gaitstratified by gender. Data are mean (standard deviation).*

|  | *Total sample*  *(n = 38)* | *Female*  *(n = 21)* | *Male*  *(n = 17)* |
| --- | --- | --- | --- |
| *Age (years)* | *73.6 (4.7)* | *73.5 (4.6)* | *73.6 (4.8)* |
| *Body weight (kg)* | *74.3 (13.1)* | *66.1 (9.6)* | *84.4 (9.3)* |
| *Body height (m)* | *1.69 (0.10)* | *1.62 (0.06)* | *1.77 (0.06)* |
| *Step length (cm)* | *64.78 (10.08)* | *60.86 (7.73)* | *69.07 (10.94)* |
| *Cadence (steps min^-1^)* | *107.5 (9.0)* | *111.1 (8.5)* | *103.0 (7.6)* |
| *Gait speed (m s^-1^)* | *1.16 (0.21)* | *1.13 (0.19)* | *1.19 (0.24)* |

8. p.11 Line 13: As a reason for the second hypothesis, could you consider whether the relatively small perturbation from the treadmill could be a factor? Discussing the potential for achieving a trunk flexion angle like overground walking by increasing the treadmill's acceleration or perturbation duration would be desirable.

[Author response] We have now added a discussion regarding the possibility of observing a greater trunk flexion with a higher treadmill acceleration as follows:

*“Treadmill perturbations with higher belt accelerations during gait (e.g. 15 m s^-2^) [24]* *may increase the initial destabilisation, forward trunk flexion, and potentially recovery step lengths compared to our findings based on a moderate acceleration (8 m s^-2^).* *However, considering the lack of swing foot obstruction, larger belt accelerations may not induce the key tripping recovery response of heightened recovery steps because there is no need to further elevate the foot.” (page 13, lines 8-13)*

We have also acknowledged that future research needs to investigate different perturbation properties (acceleration, duration, and maximum speed).

*“Fifth, the treadmill belt accelerations* *(8 m s^-2^) were delivered at the foot strike (double support stance) form but future studies may investigate different timings (e.g., mid-stance), accelerations (e.g., up to 15 m s^-2^), duration and maximum speed [26].” (page 14, lines 17-20)*

9. p.12 Line 11: Similar to the above, please discuss whether the treadmill’s perturbation is sufficient. I thought that the relatively small treadmill’s perturbation might did not increase the length nor height of recovery steps.

[Author response] We agree that larger belt acceleration magnitudes may possibly induce greater recovery step lengths in older people. However, we believe step height would not increase without physical obstacles regardless of perturbation magnitude as it is not required to elevate the perturbed foot to clear an obstacle. This is now discussed as follows:

*“Treadmill perturbations with higher belt accelerations during gait (e.g. 15 m s^-2^) [24] may increase the initial destabilisation, forward trunk flexion, and potentially recovery step lengths compared to our findings based on a moderate acceleration (8 m s^-2^). However, considering the lack of swing foot obstruction, larger belt accelerations may not induce the key tripping recovery response of heightened recovery steps because there is no need to further elevate the foot.” (page 13, lines 8-13)*

10. P12. Line 18: While a perturbation of 15 m/s^2 may indeed be excessively high, is there not a possibility that increasing it beyond 8m/s^2 could induce the treadmill walking more like overground walking? In the introduction, the authors mention the benefits of using a treadmill from a clinical application standpoint; hence, discussing how treadmill training can be optimized would offer valuable clinical implications.

[Author response] Future studies should explore the potential for treadmill belt accelerations to induce responses that better resemble obstacle trips. This has now been discussed as follows:

*“Considering the potential utility of treadmills for perturbation-based training, future research should explore the potential for treadmill belt accelerations to induce responses that better resemble obstacle trips. This may involve increasing the belt acceleration properties, adding a short deceleration prior to a rapid acceleration [24] and accelerating both belts at single stance phase (rather than at a foot-strike).” (page 13, lines 16-20)*

11. P13. Line 24: If adjusting the treadmill's perturbations can replicate the disturbances experienced during overground walking, then this expression might be considered an overestimation.

[Author response] We have modified our conclusion sentence acknowledging that only one treadmill belt acceleration setting designed for maximum destabilisation and safety was used.

*“Using maximum acceleration properties considered to be safe for older adults, our study findings suggest that kinematic responses induced by treadmill belt accelerations substantially differ from those induced by trips on a walkway in older people.” (page 14-15, lines 24-1)*

**Reviewer: 2**

Comments to the Author

12. This paper compared the kinematic responses induced by treadmill belt accelerations and those induced by trips on a walkway in older people. The results clearly indicated that the kinematic responses for the treadmill test substantially differed from those for the walkway test. The experiment was well-designed, and the paper is well-organized and well-written. However, some points need to be clear prior to publication.

[Author response] Thank you for reviewing our manuscript and providing constructive feedback to improve its clarity.

Introduction:

13. The problem statement is very clear, but the basis for the derivation of the three hypotheses listed at the end of the introduction is unclear. This point should be stated before stating the objectives.

[Author response] We have now included studies that gave rise to our three hypotheses.

The first and second hypotheses were derived from the following findings:

*“Treadmill belt accelerations have been associated with reduced dynamic stability during the first recovery step [14] and sudden forward trunk flexion [24], which are also seen following obstacle-induced trip responses [26]; [29].” (page 5, lines 6-8)*

The third hypothesis is derived from the findings below:

*“However, since treadmill belt accelerations do not obstruct the swing foot, the stepping kinematics may be different from genuine trip responses which involve elevating or lowering the tripped foot to clear the obstacle [8].” (page 5, lines 8-10)*

14. Please explain why the authors think that the acceleration on the supporting leg in treadmill walking can simulate tripping and trip-induced falls. In this case, the supporting leg moves backward, which seems to simulate a "backward slip" situation, in which the leg slips backward and forward-falls will occur. The rationale for thinking that this can simulate a trip-induced fall rather than a slip-induced forward fall must be presented.

[Author response] We agree that treadmill belt accelerations can accurately simulate forward slips at a foot-strike or backward slips at late stance phase, and that simulating trips which occur during a swing is inherently difficult. However, some previous studies have reported that accelerating the stance limb on the treadmill induces a sudden forward trunk flexion similar to a trip response. This has now been stated as follows:

*“Treadmill belt accelerations have been associated with reduced dynamic stability during the first recovery step [14] and sudden forward trunk flexion [24], which are also seen following obstacle-induced trip responses [26]; [29].” (page 5, lines 6-8)*

Methods:

15. Please show the rationale behind setting the treadmill belt acceleration magnitude and duration (8ms-2 to a maximum of 200% of usual walking speed for 30% of stride time). Can this acceleration conditions simulate the collision of swing foot?

[Author response] Considering that 15 m s^-2^ was used by Sessoms et al., 2014, in participants aged an average of 24 years, our middle-aged pilot participant reported muscle strain with 12 m s^-2^, we selected 8 m s^-2^ as the maximum acceleration which was safe for older adults. This has now been included in our methods as follows:

*“Our perturbation magnitude was determined following a pilot session with a middle-aged participant who reported muscle strain with a treadmill belt acceleration at 12 m s^-2^ to a maximum of 200% of usual walking speed for 30% of stride time.” (page 8, lines 4-7)*

It is an inherent limitation of treadmill belt accelerations that the collision of the swing foot cannot be replicated. This was a major impetus for the conduct of this study. This is now stated in the introduction as follows:

*“However, since treadmill belt accelerations do not obstruct the swing foot, the stepping kinematics may be different from genuine trip responses which involve elevating or lowering the tripped foot to clear the obstacle [8].” (page 5, lines 8-10)*

16. What is the definition of AP COM velocity on treadmill walking? Is this an AP COM velocity relative to treadmill belt velocity? Please clarify this.

[Author response] Yes, we defined anteroposterior (AP) velocity of *CoM* on the treadmill as the AP velocity of the *COM* relative to the treadmill belt velocity. This is now clarified in the methods as follows:

*“The maximum AP velocity of the CoM (m s^-1^) during each step was calculated relative to the treadmill belt velocity or ground.” (page 9, lines 4-5)*

This is also detailed in Supplementary Table 1 | Definitions of biomechanical parameters related to the treadmill and walkway.

17. In line 15 on page 8, BOS may be missing after “within the”.

[Author response] Thank you for bringing this to our attention. We have now added the missing “BoS” in the sentence.

*“[…] extrapolated centre of mass (xCoM) is within the BoS […]” (page 8, lines 22-23)*

Results:

18. Were the elevating and lowering strategy seen in treadmill walking trials? Are these strategies applicable only to walkway trials? Please clarify.

[Author response] These strategies were only applicable to walkway trip trials. This has now been clarified as follows:

*“Treadmill accelerations resulted in a 0% fall rate (n/person-trial) (note, the elevating and lowering strategies were not applicable).” (page 10, lines 11-12)*

19. The step length was reduced, and the cadence was increased for the treadmill walking. Why did these differences in gait parameters occur, and did the differences in these gait parameters affect the differences in kinematic responses between treadmill walking and over ground walking?

[Author response] We have now discussed the differences in step length and cadence and their potential impact on our study findings as follows:

*“In line with similar studies, our sample of older people had shorter step lengths and higher cadence while walking on the treadmill [41];[42]. This may reflect a “cautious gait” [43] in response to walking on an elevated, moving treadmill surface. However, we confirmed that the margin of stability and gait speed during unperturbed walking on the walkway and treadmill did not differ. Therefore, these gait parameters are unlikely to have contributed significantly to the observed differences in kinematic responses induced by the treadmill belt accelerations and walkway trips.” (page 12-13, lines 22-2)*
